# The role of the gut microbiota in shaping the tumor microenvironment and immunotherapy of breast cancer

**DOI:** 10.3389/fmicb.2025.1591745

**Published:** 2025-05-27

**Authors:** Xiaoying Ren, Lixiang Zheng, Ling Huang, Jiangbo Zhao

**Affiliations:** ^1^College of Traditional Chinese Medicine, Jiangxi University of Chinese Medicine, Nanchang, China; ^2^Jiangxi Provincial Children’s Hospital, Nanchang, China; ^3^The Affiliated Children’s Hospital of Nanchang Medical College, Nanchang, China

**Keywords:** breast cancer, tumor microenvironment, immunotherapy, gut microbiota, dietary intervention, fecal microbiota transplantation, prebiotics

## Abstract

Breast cancer is the most prevalent malignancy among women worldwide and is a major contributor to cancer-related mortality. The tumor microenvironment (TME), composed of tumor cells, immune infiltrates, fibroblasts, and vascular components, is critically involved in tumor initiation, metastatic progression, and therapeutic response. In recent years, therapies targeting the TME have undergone rapid advancements, with the objective of enhancing antitumor immunity. Concurrently, mounting evidence underscores the pivotal role of the gut microbiota and its metabolites in modulating host immunity, influencing metabolic homeostasis, inflammation, and immune equilibrium. The composition and diversity of the gut microbiome influence breast cancer progression and patients’ responses to immunotherapy. Therefore, modulating the gut microbiota is a promising strategy to enhance the clinical outcomes of TME-targeted immunotherapies. In this review, we discuss the influence of gut microbiota and its derived metabolites on breast cancer progression and immunotherapy prognosis and explore potential strategies to optimize immunotherapy through gut microbiota modulation.

## Graphical abstract

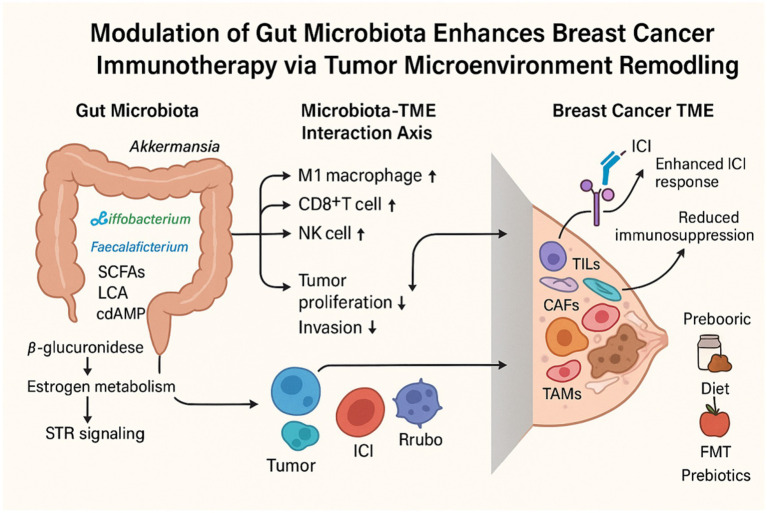

## 1 Introduction

Breast cancer represents a significant global health challenge, impacting millions of individuals each year. Epidemiological data indicate consistently high incidence and mortality rates worldwide, making it the most prevalent malignancy among women and a leading cause of cancer-related deaths (Sung et al., 2021). The management of breast cancer imposes substantial socioeconomic and healthcare burdens globally, with diagnosis, treatment, and long-term care placing significant strain on healthcare infrastructures and households alike. There is an urgent need for innovative diagnostic and therapeutic approaches to improve therapeutic efficacy and prolong patient survival (Sung et al., 2021). These strategies must integrate innovations in early detection, personalized treatment regimens, and comprehensive supportive care services to address the diverse needs of breast cancer patients worldwide.

The tumor microenvironment (TME) is a complex and dynamic ecosystem composed of malignant cells, adjacent non-malignant cells, and the extracellular matrix (ECM). It is critically involved in tumor development, metastasis, and therapeutic responses (Jin and Jin, 2020; Deepak et al., 2020). The TME encompasses cancer cells, stromal cells, vasculature, nerve fibers, the ECM, and associated acellular components, which collectively engage in complex interactions with malignant cells. These interactions regulate key oncogenic processes, including tumor proliferation, angiogenesis, evasion of apoptosis, immune suppression, and escape from immune surveillance (Hanahan and Weinberg, 2011). Given its critical role in tumor progression and therapeutic modulation, therapeutic strategies targeting the TME have witnessed significant advancements in recent years. These approaches primarily aim to modulate tumor-associated macrophages (TAMs), dendritic cells (DCs), T cells, tumor vasculature, the ECM, and cancer-associated fibroblasts (CAFs) (Bejarano et al., 2021).

The gut microbiome produces a vast array of small molecules and metabolites that are essential for various physiological processes, including metabolism, inflammation, immune regulation, and neural function (Jugder et al., 2021; Zheng et al., 2020; Erny et al., 2021; Morais et al., 2021). Given its capacity to modulate both local and systemic immune responses, there is growing interest in the influence of gut microbiota on the immune system in breast cancer and its impact on responses to immune checkpoint inhibitors (ICIs) (He et al., 2021; Fehervari, 2021; McQuade et al., 2019). Dysbiosis of the gut microbiota has also been implicated as a key contributor to cancer progression and variability in responses to ICI therapy (Mallott and Amato, 2021). Consequently, the therapeutic potential of targeting the gut microbiome as a modulator of the TME is increasingly recognized.

In this review, we provide an overview of recent advances in understanding the gut microbiota’s role in targeting the tumor microenvironment and modulating immune responses. Our aim is to elucidate the intricate relationship between the composition and function of the gut microbiome and breast cancer progression, metastasis, and therapeutic response, with an emphasis on their dynamic interplay. This understanding offers new insights into the development of microbiota-based targeted interventions to improve breast cancer treatment outcomes.

## 2 TME and immunotherapy in breast cancer

Clinically, the treatment and prognosis of breast cancer are largely determined by its molecular subtype, which is defined by the expression profiles of human epidermal growth factor receptor 2 (HER2) and hormone receptors (Prat et al., 2010; Perou et al., 2000). In recent years, therapeutic strategies such as ICI have shown considerable promise by modulating the TME instead of directly attacking tumor cells. A comprehensive understanding of the complex cellular and molecular interactions within the TME is essential for optimizing the efficacy of immunotherapeutic interventions.

### 2.1 Composition of the TME in breast cancer

TME is a highly heterogeneous and dynamically evolving milieu comprising immune cells, fibroblasts, vascular endothelial cells, and tumor-associated stroma. Different cell types within the tumor microenvironment (TME) engage in intricate crosstalk through the secretion and expression of factors that either drive tumor proliferation and immune evasion or, alternatively, support adaptive immune responses to suppress tumor growth, thereby modulating disease progression (Swanton et al., 2024).

#### 2.1.1 Macrophages

Tumor-associated macrophages (TAMs) play a pivotal role in the breast cancer TME, comprising up to 25% of immune cells in untreated triple-negative breast cancer (TNBC) (Keren et al., 2018). Within tumors, TAMs do not fit neatly into the classical dichotomy of pro-inflammatory M1 and immunosuppressive M2 macrophages; instead, they often exhibit a spectrum of polarization states, exhibiting concurrent immune-activating and immune-suppressive properties (Cassetta et al., 2019). Notably, FOLR2^+^ macrophages are positively associated with CD8^+^ T cell-mediated antitumor activity (Nalio Ramos et al., 2022). In contrast, CADM1^+^TREM2^+^ monocyte-derived macrophages become dominant as tumors progress and are strongly associated with poor prognosis in TNBC (Nalio Ramos et al., 2022). Therapeutic strategies aimed at targeting these cellular subsets have emerged as a focal point of intensive research.

#### 2.1.2 Tumor-infiltrating lymphocytes

Tumor-infiltrating lymphocytes (TILs) within the breast cancer TME consist primarily of CD3^+^ T cells. The abundance of TILs varies significantly across breast cancer subtypes, with triple-negative breast cancer (TNBC) demonstrating the greatest TIL density at the time of diagnosis. This phenomenon correlates strongly with TNBC’s elevated tumor mutational burden, high TP53 mutation frequency, and genomic instability (Luen et al., 2016). These tissue-resident memory-like (TRM-like) cells are marked by dual expression of CD103 and CD69 and often exhibit elevated levels of PD-1, CTLA-4, TIM-3, and LAG-3, indicating a dual role in cytotoxic activity and partial functional exhaustion (Savas et al., 2018). Among CD4^+^ T cells, dysfunctional TCF1^−^ subsets are found alongside helper T cells, such as Th1 and follicular helper T cells, as well as regulatory T cells (Tregs). Although Tregs constitute a relatively minor fraction, their immunosuppressive function is strongly associated with poor clinical prognosis (Keren et al., 2018; Wu et al., 2021).

In the breast cancer TME, tumor-infiltrating B lymphocytes primarily localize within clustered aggregates and are frequently associated with the formation of tertiary lymphoid structures (TLS), which resemble secondary lymphoid organs (Danenberg et al., 2022). Tumor-infiltrating B cells are more prevalent in TNBC and HER2^+^ breast cancer, and their presence is strongly associated with enhanced immunotherapy efficacy and improved clinical outcomes (Pal et al., 2021).

#### 2.1.3 Non-immune cell

As a major component of the tumor stroma, CAFs exhibit substantial heterogeneity and play a crucial role in tumor progression by remodeling the extracellular matrix, facilitating angiogenesis, and secreting cytokines that drive cancer cell proliferation, migration, and invasion (Costa et al., 2018; Kieffer et al., 2020; Cords et al., 2023; Wu et al., 2020). The CAF-S1 subset promotes Treg survival and suppresses CD8^+^ T cell activity, whereas CAF-S4 is associated with an immune-activating phenotype (Costa et al., 2018). Vascular endothelial cells regulate the recruitment and infiltration of immune cells; high endothelial venules (HEVs) enhance antitumor immunity, while FASL expression may induce apoptosis of CD8^+^ T cells (Motz et al., 2014; Goveia et al., 2020; Geldhof et al., 2022). Although often underrepresented in single-cell analyses, adipocytes exert immunomodulatory effects in the TME by secreting bioactive mediators.

### 2.2 Targeting TME

#### 2.2.1 Immune checkpoint blockade

Recent advances have achieved considerable success in immune checkpoint blockade (ICB) therapy targeting PD-1 and PD-L1 in breast cancer. The KEYNOTE trials have shown that incorporating immune checkpoint inhibitors into chemotherapy regimens markedly enhances recurrence-free survival (Cortes et al., 2022; Schmid et al., 2020). However, the heterogeneity of PD-L1 expression and interpatient variability in therapeutic response constrain the generalizability of this strategy and highlight the pressing need for reliable predictive biomarkers.

#### 2.2.2 Targeting TAMs

Recent studies have investigated approaches to modulate TAMs through key immunosuppressive pathways, such as inhibiting TREM2 or CSF1R, to enhance antitumor immune responses (Haishan Lin et al., 2008; Timperi et al., 2022). However, a key challenge is the selective depletion or reprogramming of tumor-promoting TAMs while preserving the essential functions of resident macrophages in normal tissues. Overcoming these obstacles is a central focus of ongoing research efforts.

#### 2.2.3 Targeting CAFs

Emerging therapeutic strategies aim to selectively target tumor-promoting CAFs by exploiting fibroblast activation protein (FAP) or other specific markers to alleviate tumor-induced immunosuppression within the TME (Ao et al., 2015). However, the inherent heterogeneity of CAFs and their overlapping characteristics with normal fibroblasts present major obstacles.

#### 2.2.4 Targeting angiogenesis

Therapeutic strategies targeting tumor vasculature are primarily directed at normalizing dysfunctional tumor vasculature through agents such as anti-vascular endothelial growth factor (VEGF) therapies. This approach aims to facilitate immune cell infiltration and optimize drug delivery within the TME. However, the therapeutic window for vascular normalization is relatively narrow, and responses to anti-angiogenic treatments exhibit considerable interpatient variability.

## 3 TME of breast cancer and gut microbiome

Approximately 10^14^ microorganisms inhabit the gastrointestinal tract, playing essential roles in host physiology and maintaining dynamic interactions with the host (Domenica Rea et al., 2018). Breast cancer development and progression are shaped by a multitude of factors, including age, hormonal levels, menopausal status, inflammation, and immune regulation (Wu et al., 2022). The gut microbiota plays a pivotal role in the crosstalk between these risk factors and breast cancer, influencing both tumor staging and patient prognosis (Sommer et al., 2017). Accumulating evidence indicates that the gut microbiome modulates tumor cells and their microenvironment through multiple mechanisms, including immune system modulation, alterations in estrogen metabolism, and the production of bacterial metabolites. These findings underscore the microbiota’s role in breast cancer pathophysiology and highlight its potential as a therapeutic target.

### 3.1 Estrogen metabolism and gut microbiota in breast cancer

Breast cancer, particularly hormone receptor-positive (HR^+^) subtypes, is highly dependent on estrogen signaling (Russo, 2006). Estrogen is metabolized in the liver by the cytochrome P450 enzyme system into various metabolites, which are then excreted into the intestine through bile secretion. Within the gut, specific microbial taxa—such as *Clostridia*, *Ruminococcaceae* families, and *Escherichia/Shigella* species—produce *β*-glucuronidase, an enzyme responsible for deconjugating estrogen metabolites and restoring them to their biologically active free form. These free estrogens are then reabsorbed into circulation through the portal circulation, reaching multiple organs, including the breast (Zhu and Conney, 1998; Cullin et al., 2021). This enterohepatic recycling process, known as the estrobolome, is essential in regulating systemic free estrogen levels, thereby influencing breast cancer risk and progression.

The ratio of estrogen metabolites to free estrogen exhibits an inverse correlation with breast cancer risk (Falk et al., 2013), and the gut microbiota plays a crucial role in regulating systemic estrogen levels. Gut microbial dysbiosis can lead to increased circulating estrogen levels, thereby elevating the risk of postmenopausal breast cancer. The deconjugation of estrogen in circulation is largely dependent on the composition and diversity of the gut microbiome, underscoring its mechanistic role in estrogen metabolism (Fuhrman et al., 2012). Long-term antibiotic use may disrupt the normal microbial balance, resulting in excessive *β*-glucuronidase activity. This, in turn, increases free estrogen concentrations, thereby promoting breast cancer progression (Iizumi et al., 2017). Additionally, increased adipose tissue, as seen in obesity, may also modulate estrogen levels via similar mechanisms, further heightening breast cancer risk (Keum et al., 2015). Although existing studies have established a link between estrogen metabolism and gut microbiota, the specific microbial species that play dominant roles in estrogen processing, as well as their intricate interactions, are not yet fully elucidated. The development of clinical strategies aimed at modulating the gut microbiota to regulate estrogen levels and ultimately prevent or treat breast cancer remains in its nascent stages.

### 3.2 Impact of gut microbiota on immunity

In recent years, numerous studies have revealed a complex and bidirectional regulatory interplay between the gut microbiota and the host immune system, a dynamic interaction that plays a crucial role in the initiation and progression of breast cancer. When the gut microbiota maintains homeostasis, it contributes to tissue equilibrium and suppresses tumorigenesis by modulating both innate and adaptive immune responses (Dethlefsen et al., 2007). Specifically, dysbiosis triggers the upregulation of inflammatory mediators such as IL-6 and TNF-*α*, creating a pro-inflammatory milieu conducive to breast cancer cell proliferation and invasion (Willing et al., 2011). Moreover, a reduction in gut microbial diversity is strongly associated with reduced survival outcomes in breast cancer patients, often accompanied by alterations in the immune cell compartment, further underscoring the microbiota’s role in shaping tumor immunology (Bernardo et al., 2023).

In mouse models of hormone receptor-positive (HR^+^) breast cancer, antibiotic-induced dysbiosis induces an expansion of tumor-infiltrating myeloid cells, particularly those expressing high levels of arginase-1, while also significantly promoting the infiltration of M2-like macrophages (Buchta Rosean et al., 2019). As potent immunosuppressive cells, M2-like macrophages are closely linked to immune suppression and adverse clinical outcomes (Gwak et al., 2015). Beyond macrophages, the gut microbiota plays a pivotal role in modulating T cell recruitment and activation. For instance, *Fusobacterium nucleatum* can translocate to breast tissue via the bloodstream and selectively colonize tumor sites through Fap2-dependent interactions with cancer cells. This targeted colonization inhibits the infiltration of TILs and facilitates tumor progression and metastatic dissemination (Parhi et al., 2020). Nevertheless, the precise alterations in immune cell subpopulations across different breast cancer molecular subtypes and their causal relationship with microbiota regulation are not yet well characterized. A major challenge for future therapeutic strategies is identifying optimal strategies for microbiota modulation to restore or enhance antitumor immunity.

### 3.3 The influence of gut metabolites on breast cancer

Microbial metabolites systemically distributed within the host play a pivotal role in breast cancer progression. These metabolites are either directly produced through microbial fermentation—such as short-chain fatty acids (SCFAs), lactate, and pyruvate—or result from microbial biotransformation of host-derived molecules, including secondary bile acids, aromatic hydrocarbon metabolites, and redox-modified steroids (Zhang et al., 2021). These bioactive molecules modulate host gene expression and intracellular signaling cascades. Studies have shown that early-stage breast cancer patients exhibit reduced serum levels of lithocholic acid (LCA), implicating LCA deficiency as a potential contributor to tumor progression (Miko et al., 2018). Secondary bile acids, particularly LCA, produced by *Clostridia* spp., have demonstrated inhibitory effects on breast cancer cell proliferation by activating the G protein-coupled receptor TGR5. Additionally, LCA perturbs intracellular redox equilibrium by altering the balance of antioxidant and pro-oxidant enzymes (Sampsell et al., 2020). SCFAs—such as butyrate, propionate, and acetate—constitute major microbial fermentation products of dietary fiber, primarily generated by specific gut bacterial taxa, including *Eubacterium rectale*, *Clostridium leptum*, and *Faecalibacterium prausnitzii* (Jaye et al., 2022). SCFAs are essential for regulating cellular energy metabolism, inducing cell cycle arrest, and promoting apoptosis in breast cancer cells. Notably, butyrate has been shown to independently suppress breast cancer cell growth and, when combined with anti-HER2 monoclonal antibodies such as trastuzumab, synergistically enhances therapeutic efficacy (Garmpis et al., 2017; Chen et al., 2011). While no definitive tumor-promoting microbial metabolites have been identified in breast cancer, emerging evidence suggests that microbial-derived metabolites play dual roles in tumor pathophysiology. On one hand, they directly modulate cancer cell proliferation and apoptosis. Certain microbial metabolites, such as trimethylamine N-oxide (TMAO), have been recognized for their antitumor properties. TMAO enhances CD8^+^ T cell–mediated antitumor immunity and induces ferroptosis in tumor cells, thereby contributing to tumor suppression (Dovrolis et al., 2024). On the other, they shape the local immune microenvironment, indirectly influencing tumor progression. For example, lactate accumulation within the tumor microenvironment has been strongly linked to immunosuppressive effects. Acting as a signaling molecule, lactate signals through G protein–coupled receptors (GPRs) via both autocrine and paracrine pathways, leading to enhanced angiogenesis and immune evasion in the tumor microenvironment (Zhou et al., 2024). A key challenge involves accurately characterizing and quantifying these metabolites in breast cancer patients, elucidating their spatial and temporal distribution. Furthermore, the mechanisms by which interspecies microbial interactions shape the metabolic landscape remain largely undefined. Translating these molecular insights into clinical interventions—such as targeted probiotics, prebiotics, or metabolic modulators—necessitates rigorous validation in prospective clinical studies.

### 3.4 The influence of tissue-resident microbiota within the breast tumor microenvironment

Emerging evidence indicates that tumor-resident microbiota (TRM) within breast cancer tissues play a crucial role in modulating the efficacy of immunotherapy (Ma et al., 2021). TRM may colonize tumors through migration from adjacent tissues or by translocating across compromised vasculature during systemic circulation (Fu et al., 2022). The immunosuppressive nature of the tumor microenvironment (TME) creates a permissive niche for these microbial communities (Nejman et al., 2020). Through interactions with pattern recognition receptors such as Toll-like receptors (TLRs) and G protein–coupled receptors (GPCRs), TRM regulate T cell activation and differentiation, thereby influencing therapeutic outcomes. Notably, bacterial eradication using Au–Ag₂Se nanoassemblies, in conjunction with multimodal antitumor therapy, has been shown to restore immune surveillance and potentiate antitumor immunity, resulting in recurrence-free survival extending beyond 700 days (Wang et al., 2024). However, studies targeting TRM in combination with immunotherapy remain scarce, and the precise impact of TRM on breast cancer immunotherapeutic efficacy warrants further investigation. Elucidating the composition, functional roles, and immunomodulatory mechanisms of TRM may inform the development of novel therapeutic strategies aimed at refining and enhancing breast cancer immunotherapy.

## 4 The impact of gut microbiota on immunotherapy in breast cancer

The variability in breast cancer patients’ responses to immunotherapy has posed a substantial challenge to the broad implementation of these therapies. Given the profound impact of the gut microbiota on breast cancer prognosis, it represents a key factor in personalizing immunotherapeutic strategies (Nandi et al., 2023). Emerging evidence suggests that the gut microbiome plays a pivotal role in modulating immunotherapeutic efficacy by engaging in complex interactions with the host immune system, modulating systemic inflammation, and shaping immune cell function. High-throughput sequencing and metabolomic analyses have revealed pronounced gut microbiota dysbiosis in breast cancer patients, a phenomenon closely linked to alterations in the distribution and activity of immune cells within the tumor microenvironment. These findings offer a strong scientific basis for advancing microbiota-targeted immunotherapeutic approaches in breast cancer. Table 1 summarizes the known microbial species implicated in modulating the response to immunotherapy in breast cancer.

**Table 1:** Impact of key microbial species on immunotherapeutic response in breast cancer.

Microbial species	Impact
*Faecalibacterium prausnitzii*	Produces butyrate with anti-inflammatory properties, potentially enhancing the efficacy of ICIs
*Akkermansia muciniphila*	Associated with favorable response to ICI therapy, potentially enhancing treatment efficacy by augmenting antitumor immune responses
*Bifidobacterium longum*	Associated with patients exhibiting favorable responses to ICI therapy, potentially enhancing therapeutic efficacy through modulation of the immune system
*Enterococcus hirae*	Stimulates antigen-specific immune responses and enhances antitumor immunity
*Barnesiella intestinihominis*	Activates the immune system and potentiates antitumor immune responses
*Lactobacillus* genus	Produces lactate, modulates the immune system, and enhances antitumor immune responses
*Clostridiales* order	Produces short-chain fatty acids, modulates the immune system, and enhances antitumor immune responses

In breast cancer immunotherapy, Toll-like receptors (TLRs) have emerged as key mediators that bridge innate and adaptive immune responses. Among them, TLR5 is expressed in breast cancer cells and exerts antitumor effects via the MyD88-dependent NF-κB signaling pathway. Activation of TLR5 induces the secretion of proinflammatory cytokines such as IL-8 and TNF-*α*, which subsequently suppresses tumor cell proliferation and migration (Chen et al., 2022). The expression level of TLR5 has been strongly correlated with tumor aggressiveness and patient prognosis. Downregulation of TLR5 has been shown to promote proliferation, metastasis, and epithelial-to-mesenchymal transition (EMT) in triple-negative breast cancer (TNBC), whereas high TLR5 expression is linked to diminished metastatic potential and favorable clinical outcomes (Shi et al., 2019). Moreover, TLR5 activation has been reported to induce the expression of the autophagy-associated protein MAP1S, thereby promoting autophagic activity that reinforces tumor suppression (Shi et al., 2014). The gut microbiota potentiates antitumor immune responses through Toll-like receptor (TLR) upregulation and downstream activation of inflammatory signaling pathways, thereby promoting T and B cell activation and improving the tumor immune microenvironment (Routy et al., 2018). For instance, *Salmonella typhimurium* flagellin has been shown to activate the TLR5 signaling pathway, triggering a pro-inflammatory cascade that suppresses breast cancer cell proliferation while promoting immune cell recruitment and activation, thereby augmenting immunotherapeutic efficacy (Cai et al., 2011). Studies have demonstrated that in murine models of TNBC, TLR5 agonists—such as flagellin or its derivative CBLB502—markedly extend survival and elicit durable immunological memory when administered in combination with anti–PD-1 and anti–CTLA-4 antibodies. This combinatorial strategy overcomes resistance to ICIs by activating DCs and CD8^+^ T cells, thereby enhancing antitumor immune responses (Gonzalez et al., 2023). This mechanistic insight highlights a potential therapeutic avenue for breast cancer immunotherapy, suggesting that modulation of TLR-associated signaling pathways may enhance treatment efficacy.

Activation of the STING pathway exerts both direct antiproliferative effects on tumor cells and reprograms the tumor microenvironment to enhance the efficacy of ICIs (Wang et al., 2025). This pathway is primarily initiated by cytosolic DNA sensing by cyclic GMP-AMP synthase (cGAS), which catalyzes the production of cyclic GMP-AMP (cGAMP). cGAMP subsequently activates STING, leading to robust type I interferon production, maturation of dendritic cells (DCs), and enhanced antigen presentation, ultimately driving the activation and cytotoxic activity of CD8^+^ T cells (Shen et al., 2025). In addition, STING activation upregulates chemokines such as CXCL9 and CXCL10, thereby promoting T cell infiltration into the tumor microenvironment and further augmenting antitumor immunity (de Mingo Pulido et al., 2021). Notably, the gut microbiota and its metabolites have emerged as critical modulators of STING pathway activation. Recent studies have identified *Akkermansia muciniphila* as a crucial regulator of the STING signaling pathway through the production of cyclic di-AMP (cdAMP). By activating the type I interferon (IFN-I) signaling cascade, *A. muciniphila* not only reprograms macrophages toward an antitumor phenotype but also facilitates NK cell–DC interactions, thereby orchestrating a multilayered antitumor immune response (Lam et al., 2021). Certain gut microbial taxa, such as Bifidobacterium species, can activate the host STING pathway through their metabolic products, thereby inducing type I interferon production and establishing an antiviral state in the tumor microenvironment. This, in turn, promotes the activation and infiltration of CD8^+^T cells, which enhances the efficacy of ICIs (Jia et al., 2024). However, the role of STING signaling in breast cancer remains a subject of ongoing debate. Some studies suggest that, in certain contexts, activation of the STING pathway may paradoxically promote immune evasion and therapeutic resistance (Hong et al., 2022). Moreover, the clinical application of STING agonists remains limited due to poor pharmacokinetic stability, suboptimal bioavailability, and the risk of adverse effects. Consequently, the precise modulation of STING signaling to harness its antitumor potential while minimizing unintended consequences represents a critical avenue for future research.

Activation of G protein–coupled receptor (GPCR) signaling—particularly via gut microbiota–derived SCFAs—has emerged as a key mechanism for modulating the TME and potentiating immunotherapeutic efficacy (Perez Escriva et al., 2025). SCFAs such as butyrate, propionate, and acetate signal through specific GPCRs, including GPR41 (FFAR3), GPR43 (FFAR2), and GPR109A, thereby modulating T cell function, inflammatory cytokine secretion, and tumor-associated signaling pathways, all of which contribute critically to breast cancer immunotherapy (Li et al., 2025). Studies have shown that SCFAs enhance the efficacy of ICIs by activating GPR43 and GPR41 to regulate T cell differentiation, promote Treg development, and enhance the cytotoxic activity of CD8^+^ T cells (Di Modica et al., 2022). Despite these promising findings, the role of GPCR signaling in breast cancer remains contentious. Some reports suggest that GPCR activation may, in certain contexts, promote immune escape and therapeutic resistance (Al-Qadami et al., 2022). Furthermore, the clinical application of GPCR agonists is limited by challenges such as limited receptor specificity, poor bioavailability, and adverse effects. Therefore, the precise modulation of GPCR pathways to harness their antitumor potential while mitigating deleterious outcomes constitutes a critical avenue for future research.

Despite significant progress, numerous challenges remain in leveraging gut microbiota for breast cancer immunotherapeutic interventions. First, substantial heterogeneity exists in the composition and functional profiles of the gut microbiome across different breast cancer molecular subtypes, presenting a significant barrier to the clinical translation of microbiota-targeted therapies, given the considerable interpatient variability. Second, differences in immune regulatory mechanisms between animal models and human patients limit the direct applicability of preclinical findings. Additionally, exogenous factors such as antibiotic-induced microbiome disruption can compromise immunotherapeutic efficacy. Thus, strategies for restoring or modulating beneficial microbial populations without inducing adverse effects require extensive validation in large-scale prospective clinical trials and mechanistic investigations. Future research should focus on elucidating the precise molecular interactions between the gut microbiota and breast cancer immunotherapy, establishing a conceptual and mechanistic basis for the advancement of personalized, microbiota-informed immunotherapeutic strategies.

## 5 Modulating gut microbiota as a potential therapeutic approach in breast cancer treatment

Given the critical role of the gut microbiota in breast cancer progression and its impact on immunotherapy efficacy, current research is dedicated to unraveling its therapeutic implications. Modulating the gut microbiome is gaining recognition as a promising strategy to curb disease progression and improve patient prognosis, offering a potential avenue for advancing breast cancer treatment.

### 5.1 Dietary intervention

Long-term dietary habits play a crucial role in shaping the composition and activity of the gut microbiota. Studies have shown that the gut microbiome can rapidly respond to dietary changes, even over short periods (David et al., 2014). Notably, a high-fat, high-sugar diet has been linked to adverse immune therapy outcomes, whereas sufficient dietary fiber and salt consumption has been associated with improved clinical prognosis (Szczyrek et al., 2021). In postmenopausal women, a daily fiber intake exceeding 30 g is significantly associated with a reduced risk of breast cancer while simultaneously promoting the proliferation of *Bifidobacterium* and *Faecalibacterium prausnitzii*, both of which exert anti-inflammatory and antitumor effects (Chen J. et al., 2019). The ketogenic diet, characterized by high fat, moderate protein, and low carbohydrate intake, does not appear to increase circulating estrogen levels, despite its high lipid content. Notably, when implemented in combination with multiple complementary therapies, this dietary approach resulted in complete and sustained remission in a patient with end-stage metastatic ERα-positive breast cancer (Hilakivi-Clarke et al., 2022). Additionally, polyphenols can selectively inhibit pathogenic bacteria while promoting the growth of beneficial gut microbiota. In breast cancer mouse models, polyphenols have been shown to modulate gut microbiota composition and significantly inhibit tumor progression (Nandi et al., 2023). While existing data support the beneficial role of dietary and lifestyle interventions in modulating the gut microbiota, the standardization and integration of these strategies into clinical management necessitate rigorous validation and integration into clinical practice.

### 5.2 Fecal microbiota transplantation (FMT)

Fecal microbiota transplantation (FMT) involves the transfer of gut microbiota from a healthy donor to a recipient via the upper or lower gastrointestinal tract, aiming to reestablish microbial diversity and improve immunotherapeutic outcomes (Chen D. et al., 2019). An initial clinical trial has established the feasibility and safety of FMT in immunotherapy, showing promising outcomes in patients with anti-PD-1-refractory metastatic melanoma. Among 10 patients who had previously failed to respond to PD-1 blockade, three demonstrated renewed responsiveness to PD-1 blockade post-FMT. Notably, no severe immune-related adverse events (irAEs) were reported among the patients during the treatment period (Baruch et al., 2020). FMT has not yet been investigated as a therapeutic approach for breast cancer. Additional studies are needed to assess its therapeutic potential in breast cancer treatment and to determine whether microbiota modulation through FMT could enhance treatment efficacy in this setting.

### 5.3 Prebiotics and probiotics

Prebiotics are nondigestible dietary carbohydrates, including monosaccharides, polysaccharides, and fibers, that selectively promote the growth and activity of beneficial gut microbiota (Wu et al., 2022). Studies have demonstrated that prebiotics can inhibit breast cancer progression in both *in vitro* and *in vivo* models. Beyond commonly recognized prebiotics such as dietary fiber, emerging research has highlighted the gut microbiota-modulating properties of dietary phytochemicals, including polyphenols and flavonoids (Sun et al., 2018). Although the prebiotic properties of these phytochemicals require further investigation, they have been shown to promote the expansion of microbial taxa implicated in prebiotic-mediated gut health benefits. For instance, polyphenol intake has been associated with increased abundance of Bifidobacterium and Lactobacillus, as well as enhanced production of SCFAs, highlighting their significance in gut microbiota regulation. Probiotics, including species from the *Lactobacillus* and *Bifidobacterium genera*, are live microorganisms that confer health benefits to the host by actively modulating host physiological functions (Badgeley et al., 2021). Both preclinical and clinical studies have demonstrated that probiotic interventions—such as *Lactobacillus casei BL23*, *Lactobacillus plantarum A*, or a combination of *Bifidobacterium lactis Bl-04* and *Lactobacillus acidophilus* NCFM—not only reestablish gut microbial homeostasis but also exhibit significant antitumor effects (Jacouton et al., 2017; Hibberd et al., 2017). In breast cancer survivors, administration of a probiotic blend containing *Bifidobacterium longum BB536* and *Lactobacillus rhamnosus HN001* has been shown to enhance microbial diversity while substantially lowering the *Bacteroidetes*-to-*Firmicutes* ratio, an indicator of gut microbiota dysbiosis. Additionally, probiotic supplementation has been linked to decreased body mass index (BMI) and enhanced insulin sensitivity (Pellegrini et al., 2020). These findings suggest that probiotics hold promise as an adjunctive approach for regulating metabolic and immune functions in breast cancer care.

Although these strategies have shown substantial antitumor efficacy in preclinical studies, significant interpatient variability and the intrinsic heterogeneity of gut microbiota composition pose substantial challenges to their broad clinical application. Tailored strategies based on individual microbiome profiles may be essential to optimize the therapeutic efficacy of microbiota-based interventions in breast cancer treatment.

## 6 Conclusion and perspectives

The role of the gut microbiota in shaping the breast cancer tumor microenvironment has attracted growing interest. Current research indicates that the microbiota exerts both direct and indirect effects on breast cancer initiation, progression, and metastasis through its regulation of estrogen metabolism, inflammatory signaling, and immune cell function. Moreover, its composition is strongly associated with the efficacy of immune checkpoint inhibitors and other therapeutic strategies. Although preclinical studies and emerging clinical data suggest that microbiota modulation represents a promising avenue for improving immunotherapy efficacy, several challenges remain in translating these findings into clinical applications. The causal relationships among the gut microbiota, immune system, and tumor development remain incompletely defined, and the underlying mechanisms warrant further in-depth investigation. The substantial heterogeneity in microbiota composition and function across different breast cancer molecular subtypes and among individuals poses a significant challenge to 389the standardization of microbiota-targeted therapies. Additionally, the precise mechanisms by which gut microbiota modulates immune responses through pathways such as TLR and STING, as well as the spatiotemporal dynamics of these interactions within the tumor microenvironment, are not yet fully elucidated. Comprehensive mechanistic studies to bridge these knowledge gaps will be essential for advancing microbiota-based strategies in breast cancer treatment. Future research should focus on identifying reliable biomarkers that can predict which subsets of breast cancer patients are most likely to respond to microbiota-targeted interventions. Moreover, a critical challenge in translating microbiota-targeted strategies from animal models to clinical applications is ensuring targeted and safe modulation while preserving microbial homeostasis in patients. Exogenous factors such as antibiotics, dietary interventions, and other environmental influences can perturb the gut microbiota, thereby potentially compromising immunotherapeutic efficacy. Therefore, well-designed large-scale prospective clinical trials are critical for rigorously assessing the safety and effectiveness of microbiota-modulating interventions. Future research should integrate multi-omics approaches, utilizing advanced technologies, including single-cell sequencing, spatial transcriptomics, and metabolomics, to systematically decipher the intricate interactions between the gut microbiota and the tumor microenvironment. Such comprehensive analyses will establish a solid conceptual framework for advancing personalized and precision-based therapeutic strategies in breast cancer treatment. Standardized and systematic clinical trial designs are critically needed to rigorously assess the efficacy and safety of various microbiota-based interventions—such as probiotics, FMT, and targeted metabolite supplementation—across distinct breast cancer subtypes. In parallel, the development of personalized microbial therapies, including customized probiotic formulations tailored to individual microbiota profiles and immunological features, represents a promising avenue for precision medicine. However, most current evidence is largely restricted to preclinical animal models, raising critical questions about the translatability of these findings to human settings. Challenges include interspecies differences in immune architecture and microbial ecology, the stability and reproducibility of microbiota interventions in humans, and the high degree of interindividual heterogeneity in immunotherapeutic responses. Therefore, future efforts should emphasize interdisciplinary collaboration and large-scale human cohort studies to establish clinically relevant model systems and accelerate the translation of microbiome research into therapeutic applications.

Overall, modulating the gut microbiota as a potential strategy for enhancing breast cancer immunotherapy represents a promising avenue. The integration of multimodal therapeutic strategies, including dietary interventions, prebiotic/probiotic supplementation, and microbiota transplantation, in conjunction with the continuous refinement of immunotherapeutic regimens, may help address existing challenges, facilitate clinical translation, and ultimately improve patient outcomes.
